# As-grown graphene/copper nanoparticles hybrid nanostructures for enhanced intensity and stability of surface plasmon resonance

**DOI:** 10.1038/srep37190

**Published:** 2016-11-22

**Authors:** Yun-Fei Li, Feng-Xi Dong, Yang Chen, Xu-Lin Zhang, Lei Wang, Yan-Gang Bi, Zhen-Nan Tian, Yue-Feng Liu, Jing Feng, Hong-Bo Sun

**Affiliations:** 1 State Key Laboratory on Integrated Optoelectronics, College of Electronic Science and Engineering, Jilin University, 2699 Qianjin Street, Changchun, 130012, People’s Republic of China; 2 College of Physics, Jilin University, 2699 Qianjin Street, Changchun, 130012, People’s Republic of China

## Abstract

The transfer-free fabrication of the high quality graphene on the metallic nanostructures, which is highly desirable for device applications, remains a challenge. Here, we develop the transfer-free method by direct chemical vapor deposition of the graphene layers on copper (Cu) nanoparticles (NPs) to realize the hybrid nanostructures. The graphene as-grown on the Cu NPs permits full electric contact and strong interactions, which results in a strong localization of the field at the graphene/copper interface. An enhanced intensity of the localized surface plasmon resonances (LSPRs) supported by the hybrid nanostructures can be obtained, which induces a much enhanced fluorescent intensity from the dye coated hybrid nanostructures. Moreover, the graphene sheets covering completely and uniformly on the Cu NPs act as a passivation layer to protect the underlying metal surface from air oxidation. As a result, the stability of the LSPRs for the hybrid nanostructures is much enhanced compared to that of the bare Cu NPs. The transfer-free hybrid nanostructures with enhanced intensity and stability of the LSPRs will enable their much broader applications in photonics and optoelectronics.

Graphene as two-dimensional one-atom-thick sheet of carbon has shown its potential for a wide variety of applications^1^,^2^,^3^. Recently, hybrid nanostructures consisting of graphene and metallic nanostructures have attracted much attention because of the enhanced light–matter interactions in the hybrid nanostructures, which hold great promise for applications in photonics and optoelectronics^4^,^5^,^6^,^7^,^8^,^9^,^10^,^11^,^12^. Metallic nanoparticles (NPs) have shown prominent optical properties^13^,^14^,^15^ due to localized surface plasmon resonances (LSPRs) associated with the excitation of a collective oscillation of electrons^16^,^17^,^18^,^19^. An enhanced intensity of the LSPRs induced by the enhanced light–matter interactions is expectable for the hybrid nanostructures of graphene/metal NPs. Copper (Cu) has competitive advantages over the noble metals in low cost, compatibility of integrated circuit, high thermal and electrical conductivities and low electro-migration resistance^20^,^21^,^22^,^23^. However, the Cu NPs are chemically reactive and rapidly oxidized under ambient conditions^24^, which results in degradation in the LSPRs intensity^25^,^26^. Protective coatings are usually applied to guard the Cu NPs against reactive environments^27^,^28^,^29^,^30^, while the protective coating-induced changes of the optical or electrical properties of the Cu surface should be avoided. Graphene can act as an optically thin oxidation barrier to a pure unoxidized metal surface with minimized changes to the physical properties of the protected Cu^31^,^32^,^33^. Thus, simultaneous improvement in both intensity and stability of the LSPRs supported by the Cu NPs is expectable by coating graphene onto the Cu NPs to form the graphene/Cu NPs hybrid nanostructures.

The graphene/metal hybrid nanostructures are usually fabricated by separate preparation of metal nanostructures and wet etching and transferring the CVD grown graphene onto the metal nanostructures^5^,^34^,^35^,^36^. However, the etching and transferring processes generate structural and chemical deterioration in the graphene thin film^37^,^38^. It is time-consuming and inevitably requires the disposal of the metal substrates, generating hazardous chemical waste, which significantly limits the cost-competitive and environmentally friendly mass production of graphene-based devices^39^. Moreover, the incomplete contact between the graphene and metal surface impedes the graphene-induced light–matter interactions^40^. Additionally, Cu-based nanostructures are not suitable for this method since they would be etched by the ferric chloride corrosion liquid during the transferring process. So far, the transfer-free fabrication of the high quality graphene/Cu NPs hybrid nanostructures remains a challenge.

Here, we develop an etching and transfer-free technique for one-step fabrication of the graphene/Cu NPs hybrid nanostructures by direct growth of graphene on the hemispherical copper nanoparticles to form graphene-coated hemispheres with Cu cores. The as-grown graphene/Cu NPs hybrid nanostructures exhibit enhancements in both intensity and stability of the LSPRs due to the graphene-induced enhancement in the light–matter interactions and the surface passivation. The as-grown graphene on the Cu NPs is beneficial to their full electric contact and strong interactions on account of the larger adhesion energy. 10-fold enhancement of fluorescence from the dye coated hybrid nanostructures has been obtained due to graphene-induced enhanced LSPRs, which has been supported by finite-difference time domain (FDTD) calculation. Moreover, the stability of the LSPRs for the hybrid nanostructures is much enhanced compared to that of the bare Cu NPs due to the surface passivation of the graphene sheets covering completely and uniformly on the Cu NPs. Our findings might open up a new avenue to realize high quality graphene/Cu NPs hybrid nanostructures and fulfil their practical applications in photonics and optoelectronics.

## Results and Discussion

**Figure 1:**
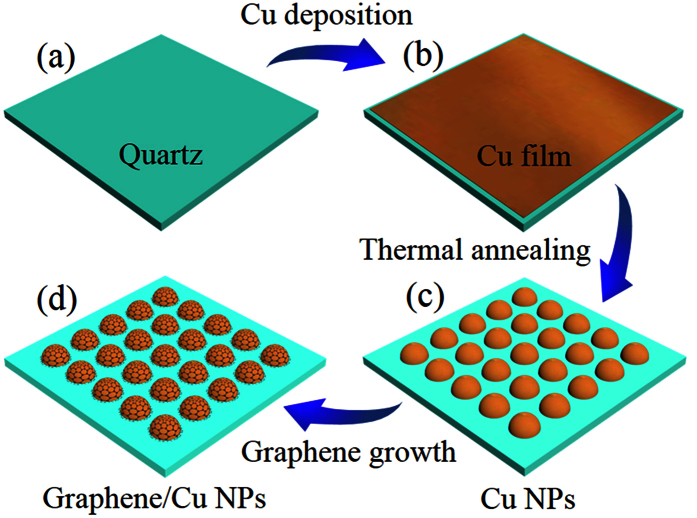
Schematic illustrations of fabrication sequences for the as-grown graphene/Cu NPs hybrid nanostructures. (**a**,**b**) Deposition of the Cu film on the quartz substrate. (**c**) Thermal annealing of the Cu film. (**d**) Deposition of the graphene on the Cu NPs.

**Figure 2:**
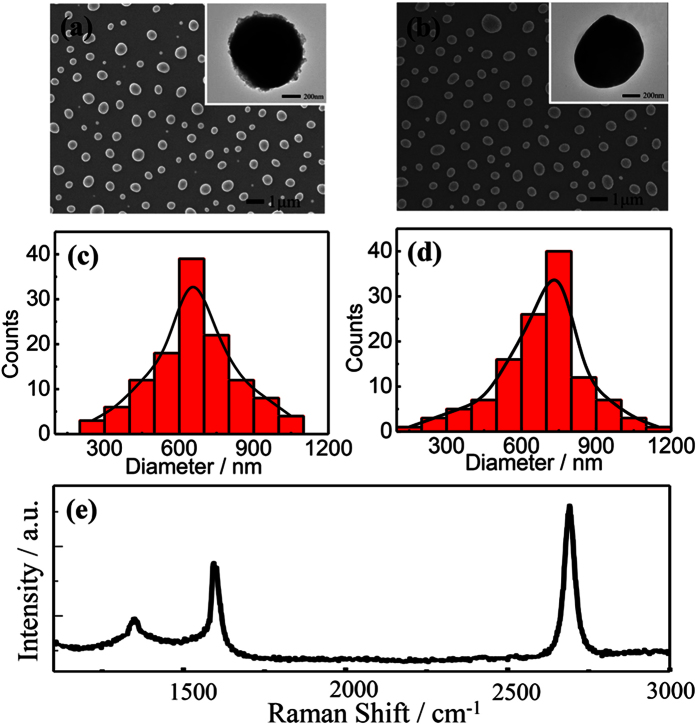
(**a**,**b**) SEM images and HRTEM images (the inset figures) of Cu NPs with and without graphene as protective layers, respectively. (**c**,**d**) Size distributions of as-grown graphene/Cu NPs hybrid nanostructures and bare Cu NPs. (**e**) Normal Raman spectrum of graphene after removing Cu cores.

The specific fabrication procedures of the as-grown graphene on the hemispherical copper nanoparticles are schematically summarized in Fig. 1 and illustrated in the experimental details. During the annealing process of high temperature, copper film melts into nanoparticles and patterned graphene films directly form on them without transfer method. The surface morphologies of the copper nanoparticles coated with and without the as-grown graphene layers are investigated by scanning electron microscopy (SEM) and high resolution transmission electron microscopy (HRTEM) and shown in Fig. 2a,b, respectively. There is no apparent morphological variation except irregular edges with the as-grown graphene. The Cu NPs with diverse size distributions mainly ranges from 600 to 800 nm (Fig. 2c,d). It has been demonstrated that the graphene encapsulation process performed on the separately prepared Cu NPs is prone to induce agglomeration at high temperature when gas carbon source such as methane or ethylene is supplied for the growth of graphene^29^,^41^. While in our case, the Cu NPs are not agglomerated during the annealing process at high temperature of 1000 °C even using methane as gas carbon source in this one-step method. This can probably be attributed to the synchronous synthesis of Cu NPs and graphene layers, where Cu NPs could be defended against morphological destruction by the as-grown protective graphene shells during the annealing procedure. To further confirm the formation and investigate the quality of graphene layers coated on the Cu NPs, we conduct typical Raman measurements using laser wavelength of 532 nm as shown in Fig. 2e. Three most intensive features associated with graphene, the D peak at 1337 cm^−1^, the G peak at 1580 cm^−1^ and the 2D peak at 2659 cm^−1^ are observed, respectively, which confirms the direct synthesis of few layer graphene on Cu NPs. In particular, the D peak at 1337 cm^−1^ originates in the lattice disorder probably arising from the etch process for analysis or the high curvature of Cu NPs surfaces, rather than the practical defects in the graphitic lattice.

**Figure 3:**
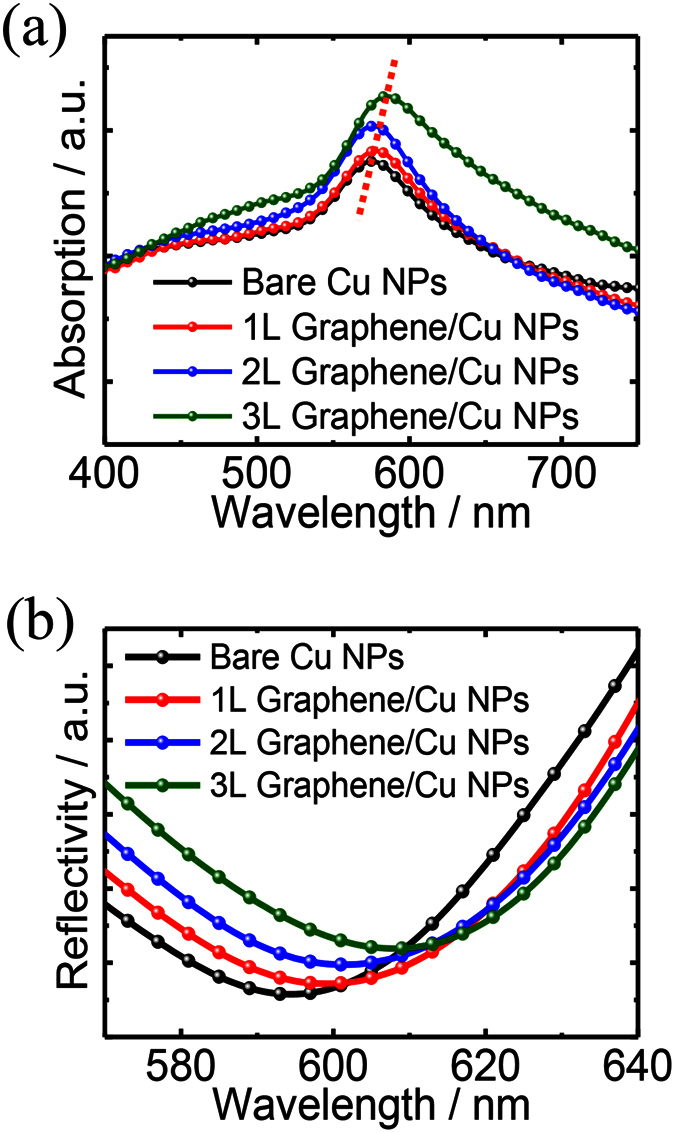
(**a**) Steady-state optical absorption spectra versus wavelength for the Cu NPs coated with various numbers of graphene layers. (**b**) Simulated reflection spectra of Cu NPs coated with various numbers of graphene layers.

To shed light on the specific function of graphene coupling to the LSPRs of the metal nanoparticles, the steady-state optical absorption spectra for the Cu NPs with or without the graphene coating are compared and shown in Fig. 3a. The layer numbers of graphene can be precisely controlled from single layer to three layers by adjusting the gas concentration and growth time. The enhanced absorption intensity can be observed with the increased layer numbers of the graphene, meanwhile, the absorption peaks redshift gradually. The presence of the graphene sheets results in a strong localization of the field at the graphene/Cu NPs interface and a drastic field enhancement, especially with full electric contact and strong interactions in the hybrid nanostructures. It has been reported that electrons would transfer from graphene to the surface of Cu thin film in order to maintain the continuity of the Fermi levels when they contact with each other, as the work function of Cu (5.22 eV) is larger than that of graphene (4.5 eV)^42^,^43^. Therefore, charge transfer is one of the main reasons for the LSPRs enhancement induced by the graphene layers^44^. Besides, the as-grown graphene on the Cu NPs is beneficial to their full electric contact and strong interactions arising from the larger adhesion energy, since the attractive intersurface forces such as van der Waals pull the graphene sheets into tight contact with the growth substrate, where an enhanced intensity of the LSPRs supported by the hybrid nanostructures can be obtained. The LSPRs are highly sensitive to the surrounding environment where graphene acts as a lossy dielectric with higher refractive index in the visible and near-infrared wavelength, which results in the red-shift of the LSPRs wavelength with the increased layer number of the graphene^45^.

In-house-generated finite-difference time-domain (FDTD) simulations are performed to further investigate the LSPRs enhancement induced by the graphene coating. We calculate the reflection spectra of the graphene-coated Cu NPs arrays versus different numbers of the graphene layers as shown in Fig. 3b, where the red-shift and low reflection can be observed with increased graphene layer numbers. It is noteworthy that we treat monolayer graphene as an effective medium with thickness of 1 nm which is reasonable for CVD-grown graphene. This shows a good agreement with the experimental results and indicates that the strong light-graphene interactions can account for the remarkable resonance shift as well as enhanced absorption between 400 nm and 750 nm^44^. More specifically, the red-shift of the resonance is a result of the introduction of the graphene layers, since besides the metal properties, the LSPRs are also determined by the refractive index of the environment. The broadband absorption enhancement is due to the fact that graphene is a good absorber in optical frequency region.

**Figure 4:**
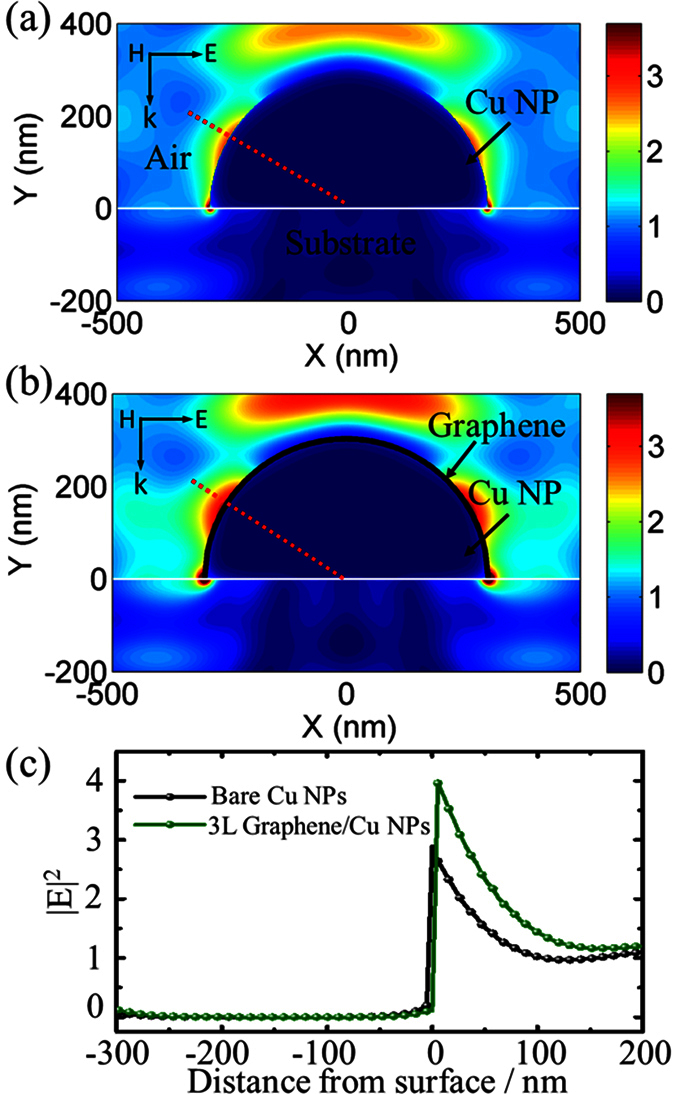
Simulated electrical field distributions for the bare Cu NP (**a**) and as-grown graphene/Cu NP hybrid nanostructure (**b**). (**c**) Cross-section plots for total electric fields of Cu NP along the red dot line in (**a**,**b**) with (green) and without (black) graphene layer, respectively.

**Figure 5:**
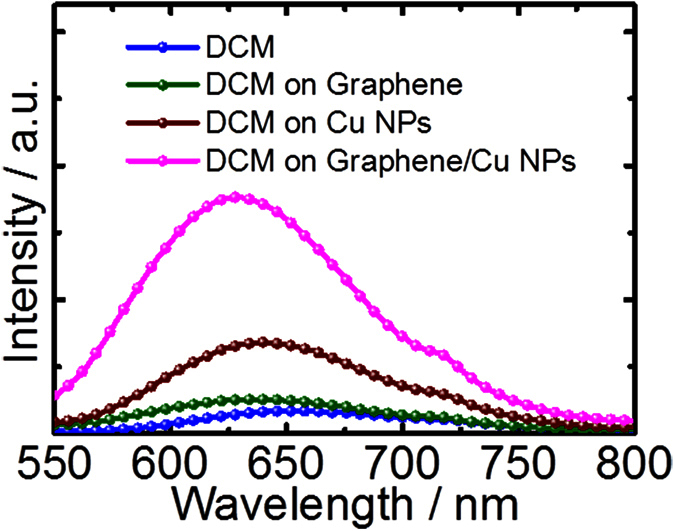
Fluorescence spectra of DCM dye on quartz substrate, graphene films, bare Cu NPs, and as-grown graphene/Cu NPs hybrid nanostructures, respectively.

Figure 4a,b illustrate the normalized electrical field distributions for the corresponding LSPRs excited in the bare Cu NP and graphene/Cu NP hybrid nanostructure with the incident light at the wavelength of 595 nm and 610 nm, respectively. The field intensity |E|^2^ exhibits its maximum at the surface of Cu NPs and shows exponential decay along the direction perpendicular to it, demonstrating the excitation of the LSPRs. In this case, the presence of graphene sheets leads to a strong localization of the field at the graphene–copper interface, as shown in Fig. 4b, when compared with the bare Cu NPs in Fig. 4a. The cross-section plots for total electric fields (Fig. 4c) indicate the obvious field enhancement for the hybrid nanostructures. LSPRs have effect on fluorescence enhancement for the fluorescent dye adsorbed on the metallic NPs through the strong coupling between the dye molecule resonance and the NPs LSPRs. Fluorescence spectra of the 4-(Dicyanomethylene)-2-methyl-6-(4-dimethylaminostyryl)-4H-pyran (DCM) dye deposited on the as-grown graphene/Cu NPs hybrid nanostructures, bare Cu NPs and graphene films, respectively, are measured and compared to examine the LSPRs enhancement. As can be seen in Fig. 5, a highest fluorescence enhancement factor of 10.0 is obtained from the hybrid nanostructures with three-layered graphene compared to the DCM on bare quartz substrate, while the enhancement factors are 4.0 and 1.7, respectively, for that on the Cu NPs and graphene films. The highest fluorescence enhancement should be attributed to the enhanced intensity of the LSPRs supported on the graphene/Cu NPs hybrid nanostructures.

**Figure 6:**
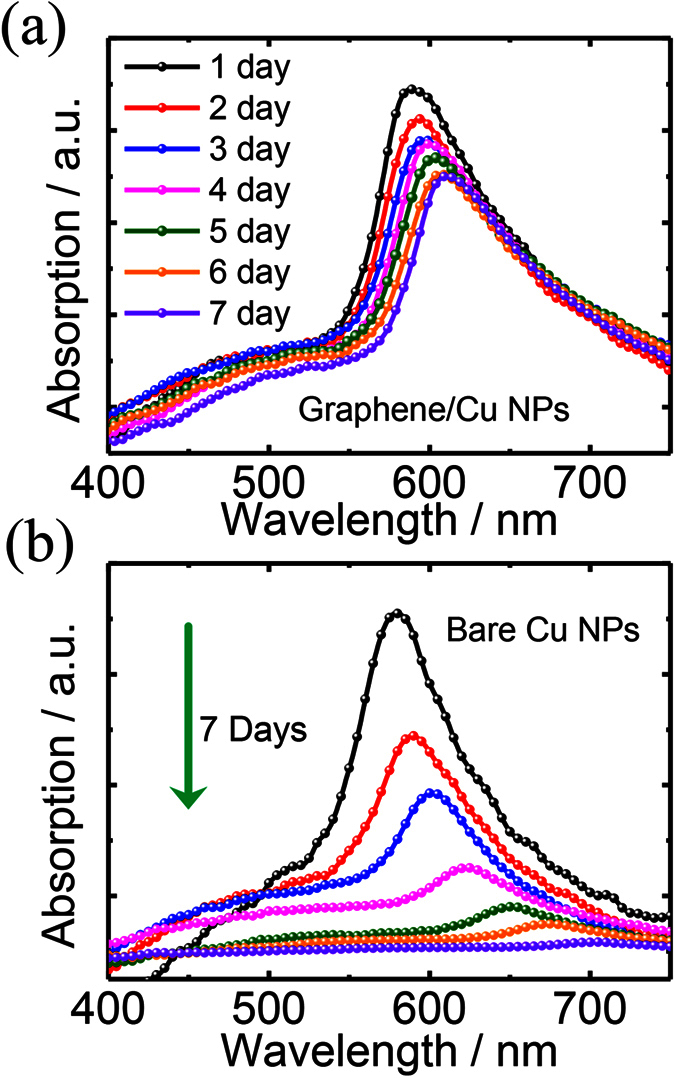
Time evolution of the absorption spectra of graphene-coated (**a**) and uncoated (**b**) Cu NPs.

The surface of the bare copper is prone to proceed oxidation as exposed to the ambient air, leading to the development of layers consisting of cuprous oxide (Cu_2_O), cupric oxide (CuO) and copper hydroxide (Cu(OH)_2_)^19^,^20^,^23^,^46^. In order to evaluate the passivation of the graphene on the Cu NPs, the UV–Vis absorption spectra during the time of exposure to ambient air for Cu NPs with or without the coating of three-layered graphene are investigated and shown in Fig. 6. For the fresh Cu NPs with or without the graphene coating, their absorption spectra show a prominent absorption peak at around 580 nm. Red-shift and degradation of the LSPRs can be observed for both bare and graphene-coated Cu NPs with the increased exposure time. However, the degradations are much smaller for the hybrid nanostructures. Especially, the absorption peak of the bare Cu NPs almost disappears after 7 days exposure, which indicates the complete oxidation of the bare Cu NPs due to their active property. While in case of the hybrid nanostructures, the much improved stability of the LSPRs verifies the effective passivation of the graphene.

**Figure 7:**
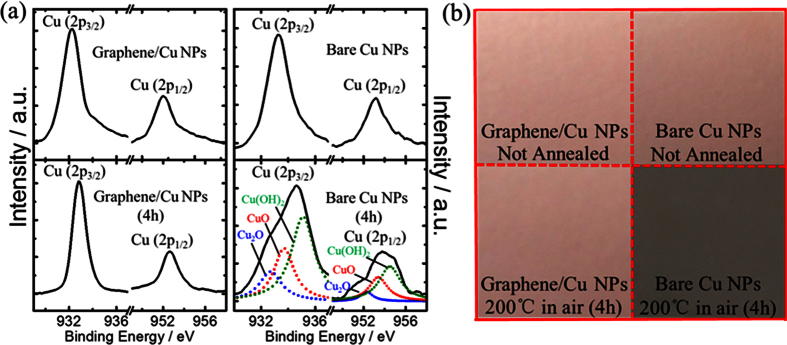
Time evolution of the XPS core-level Cu2p spectra (**a**) and photograph images (**b**) of graphene/Cu NPs hybrid nanostructures and bare Cu NPs before and after thermal treatment at 200 °C for 4 hours.

To further evaluate the graphene passivation, X-ray photoelectron spectroscopy (XPS) measurement is carried out to analyze the metal composition. Figure 7a shows the XPS spectra of bare Cu NPs and three-layered-graphene/Cu NPs hybrid nanostructures before and after thermal treatment at 200 °C for 4 hours in ambient conditions. Several studies have proposed that the surface oxidation for polycrystalline Cu proceeds in four distinct steps: (a) dissociative adsorption of O_2_, (b) initial formation of cuprous oxide (Cu_2_O), (c) formation of cupric oxide (CuO) islands, and (d) further formation of copper hydroxide (Cu(OH)_2_)^20^. Here, the XPS spectra of graphene coated Cu NPs (Fig. 7a, left) exhibit two prominent Cu peaks representing Cu 2p_3/2_ and Cu 2p_1/2_ at binding energies of 932.2 eV, 952.3 eV (before thermal treatment) and 932.8 eV, 952.5 eV (after thermal treatment), respectively, which indicates negligible oxidation of the graphene/Cu NPs hybrid nanostructures after thermal treatment. However, in case of the bare Cu NPs (Fig. 7b, right), broader peaks corresponding to different copper oxides composition, Cu_2_O (932.7 and 952.3 eV), CuO (933.7 and 953.4 eV), and Cu(OH)_2_ (935.1 and 954.5 eV) are formed after thermal treatment for 4 hours. The photograph images of both thermal treatments are shown in Fig. 7b. The graphene/Cu NPs hybrid nanostructures exhibit little visible variations, in contrast with the bare Cu NPs whose surface morphology changes remarkably. The XPS datas and photograph images combined with the absorption spectra demonstrate that the graphene coating is effective on protecting the underlying Cu NPs from oxidation.

## Conclusion

In conclusion, we have demonstrated a novel and facile route to fabricate the hybrid nanostructures of as-grown etching-free graphene on Cu NPs with full electric contact and strong interactions by the chemical vapor deposition (CVD) process. Few-layer graphene can be directly synthesized on the Cu NPs, which is verified from the Raman spectroscopy and HRTEM micrographs. The intensity of the LSPRs supported by the graphene/Cu NPs hybrid nanostructures has been improved, and results in an 10-fold enhanced fluorescent intensity from the dye coated hybrid nanostructures. The stability of the LSPRs for the hybrid nanostructures has been much enhanced compared to that of the bare Cu NPs due to the passivation of the graphene coating. The transfer-free hybrid nanostructures with enhanced intensity and stability of the LSPRs might play a significantly important role in the development of Cu-based plasmonic nanostructures, hybrid nanophotonic and optoelectronic devices and ultra-large-scale integrated circuits.

## Methods

Fabrication and characterization of graphene/Cu NPs hybrid nanostructures: The copper films of 40 nm were fabricated on the pre-cleaned quartz substrates (cleaned by acetone, ethanol and ultrapure water, respectively) via thermal evaporation with pieces of copper foil (Alfa Aesar, item No. 46356) at a base pressure of 5 × 10^−4^ Pa. Thermal annealing then preformed in the low pressure chemical vapor deposition (LPCVD) system at the atmosphere of hydrogen (H_2_) and argon (Ar) gas to obtain copper nanoparticles with morphology-controllable characteristics. After the introduction of methane (CH_4_) gas into the chamber at the proper temperature of 1000 °C, graphene/copper nanoparticles hybrid nanostructures can be realized directly after the rapid cooling without time-consuming transfer method. The gas flow rates of CH_4_, H_2_ and Ar were 7, 30, 200 standard cubic centimeters per minute (sccm), respectively. The ramping rate of the temperature was approximately 40 °C/min, followed by rapid cooling to room temperature (25 °C) with protective gas configuration of H_2_ and Ar.

Scanning electron microscopy (SEM, JEOL JSM-6700F, Japan) and high resolution transmission electron microscopy (HRTEM, JEOL JEM-2100F, Japan) were utilized to measure the surface morphologies of the samples. Raman spectra (LabRAM HR Evolution Raman Spectrometer, HORIBA Scientific, France) were obtained with a 532 nm laser to analyze the quality of graphene. In order to remove the influence of Cu fluorescence peaks overlapping with typical graphene peaks, the specimens were thoroughly soaked in 20% HNO_3_ for 10 h and rinsed in deionized water for 20 min subsequently to etch the Cu cores prior to the micro-Raman spectroscopy analysis. Absorption spectra were measured using UV-vis spectrophotometer (UV-2550, Shimadzu, Japan). The chemical composition of the specimens was characterized by X-ray photoelectron spectroscopy (XPS, ESCAL-AB250, VG Microtech, UK).

A 25 nm thick emitting layer of 4-(Dicyanomethylene)-2-methyl-6-(4-dimethylaminostyryl)-4H-pyran (DCM) was thermal evaporated on the specimens of graphene films, bare Cu NPs, graphene/Cu NPs and quartz substrates at a base pressure of 5 × 10^−4^ Pa. The fluorescence spectra were obtained at the excitation light of 480 nm from fluorescence spectrophotometer (F-4600, HITACHI, Japan).

## Additional Information

**How to cite this article**: Li, Y.-F. *et al*. As-grown graphene/copper nanoparticles hybrid nanostructures for enhanced intensity and stability of surface plasmon resonance. *Sci. Rep.* **6**, 37190; doi: 10.1038/srep37190 (2016).

**Publisher's note:** Springer Nature remains neutral with regard to jurisdictional claims in published maps and institutional affiliations.
